# Hair and stress: A pilot study of hair and cytokine balance alteration in healthy young women under major exam stress

**DOI:** 10.1371/journal.pone.0175904

**Published:** 2017-04-19

**Authors:** Eva M. J. Peters, Yvonne Müller, Wenke Snaga, Herbert Fliege, Anett Reißhauer, Thomas Schmidt-Rose, Heiner Max, Dorothea Schweiger, Matthias Rose, Johannes Kruse

**Affiliations:** 1 Universitätsmedizin Charité, Center 12 for Internal Medicine and Dermatology, Division for General Internal Medicine, Psychosomatics and Psychotherapy: Psycho-Neuro-Immunology Skin Research Group, Berlin, Germany; 2 Justus-Liebig-University, Department of Psychosomatics and Psychotherapy, Psychoneuroimmunology Laboratory, Gießen, Germany; 3 Foreign Office, Health Service, Psychosocial Counseling, Auswärtiges Amt, Berlin, Germany; 4 Universitätsmedizin Charité, Center 12 for Internal Medicine and Dermatology, Division for Physical Medicine and Rehabilitation, Berlin, Germany; 5 Beiersdorf AG, Hamburg, Germany; Universitatsklinikum Hamburg-Eppendorf, GERMANY

## Abstract

Mouse models show that experimental stress mimicking prolonged life-stress exposure enhances neurogenic inflammation, induces adaptive immunity cytokine-imbalance characterized by a shift to Type 1 T-helper cell cytokines and increases apoptosis of epithelial cells. This affects hair growth in otherwise healthy animals. In this study, we investigate whether a prolonged naturalistic life-stress exposure affects cytokine balance and hair parameters in healthy humans. 33 (18 exam, 15 comparison) female medical students with comparable sociobiological status were analyzed during a stressful final examination period, at three points in time (T) 12 weeks apart. T1 was before start of the learning period, T2 between the three-day written exam and an oral examination, and T3 after a 12 week rest and recovery from the stress of the examination period. Assessments included: self-reported distress and coping strategies (Perceived Stress Questionnaire [PSQ], Trier Inventory for the Assessment of Chronic Stress [TICS]), COPE), cytokines in supernatants of stimulated peripheral blood mononucleocytes (PBMCs), and trichogram (hair cycle and pigmentation analysis). Comparison between students participating in the final medical exam at T2 and non-exam students, revealed significantly higher stress perception in exam students. Time-wise comparison revealed that stress level, TH1/TH2 cytokine balance and hair parameters changed significantly from T1 to T2 in the exam group, but not the control. However, no group differences were found for cytokine balance or hair parameters at T2. The study concludes that in humans, naturalistic stress, as perceived during participation in a major medical exam, has the potential to shift the immune response to TH1 and transiently hamper hair growth, but these changes stay within a physiological range. Findings are instructive for patients suffering from hair loss in times of high stress. Replication in larger and more diverse sample populations is required, to assess suitability of trichogram analysis as biological outcome for stress studies.

## Introduction

Attempts to define clinical outcomes for the study of deleterious psycho-social stress have occupied multiple researchers over the past decades [1–3]. The skin contains a mini-organ, the hair follicle (HF), which is subjected to life-long remodeling via the hair cycle. Experimental animal models suggest that perceived stress can trigger neuroendocrine-immune changes, which interfere with the organism’s tissue regenerative capacity such that in young female mice, prolonged noise- or restraint stress paradigms provoke neurogenic inflammation and subsequent HF regression (catagen).

In humans, hair loss is often reported clinically during periods of excessive stress [4–6]. Hair loss may indicate underlying organic disease, however, frequently no organic disturbances can be found, which can be disturbing for hair loss patients and their doctors. It was therefor hypothesized that analagous to the animal models, in humans, stress activates neuroendocrine-immune circuits [7], which terminate hair growth in the absence of other clincally noticable health disturbances [8, 9]. Such circuits in mice involve the dense peptidergic innervation of organs at the self-environment interface (e.g. skin, gut, lungs) and its interaction with mast cells [10]. The subsequent activation of the later is a common mechanism in response to a wide variety of stressors, including psycho-social stress. In the wake of stressful events, cytokines which regulate innate and adaptive immune responses are released. These include tumor necrosis factor alpha (TNFα) or interferon gamma (IFNγ) [2]. Under conditions of stress they participate in cellular adaptive immune responses traditionally addressed as T-helper cell Type 1 (TH1). As a consequence, in response to prolonged stress-exposure in otherwise healthy animals, epithelial and mesenchymal cells in the skin are driven into apoptosis [11, 12] or senescence [13] and premature termination of hair growth occurs [14, 15].

There is a large body of research relating to stress and inflammation in animals and humans, that extensively examines cytokines and other pro-inflammatory effects of stress [1, 2, 8, 16–18]. Only a small number of human studies have addressed the association between stress, immune response and tissue regeneration in healthy, non-wounded humans, under prolonged, real life-stress exposure [16, 19–22]. As a result, no scientific evidence for stress-induced hair loss in humans is available to date. Hair analysis as an indicator for stress in studies of human subjects therefor remains to be explored. In an attempt to fill this gap and relate previous mouse studies to humans, we investigated the proposed hypothesis through trichogram analysis in a pilot study. The sample population was designed to match the experimental set-up previously reported in murine studies on stress and hair: healthy, young, sociobiologically similar females under prolonged stress-exposure [23]. Specifically, we asked if prolonged life-stress exposure, induced by the preparation for and participation in the final medical exam, had the capacity to induce premature HF regression in female medical students, as compared to points in time outside the exam-period or to non-exam students.

To test this hypothesis, we opted for a non-invasive sampling approach (trichogram). This established an ethical and sustainable method suitable for field studies. Further, we assessed the suitability of the ‘Perceived Stress Questionnaire’ (PSQ), the ‘Trier Inventory for the Assessment of Chronic Stress’ (TICS) and the ‘COPE’ for the assessment of stress perception and coping strategies in this context, and of peripheral blood mononucleocyte (PBMC) cytokine production for the assessment of changes in cytokine balance between T-helper cell Type 1 (TH1) cytokines (TNFα, IFNγ) and TH2 cytokines (interleukin 4 [IL4], IL5). Using this quasi experimental, naturalistic approach, we partially confirmed our hypothesis.

## Materials and methods

### Ethics and recruitment of participants

The study was conducted in accordance with the Declaration of Helsinki and approved by the ethics committee of the Charité-Universitätsmedizin, Berlin, Germany and the ethics committee of the Justus-Liebig University, Gießen, Germany. For this pilot study, a homogeneous sample was recruited to meet the ethical requirement to keep the pilot study sample as small as possible. Participants were recruited through an advertisement on a digital university notice board. After a telephone interview, to clarify inclusion and exclusion criteria, participants who provided written informed consent were enrolled.

### Inclusion and exclusion criteria

The study used the following inclusion and exclusion criteria: Caucasian female, medical student, aged between 18 and 48; all hair colors except for gray; hair length long enough to pluck (at least 2 cm); no haircuts or hair dye in the week prior to first examination or during the observation period; no hormonal or endocrine oral medication including corticosteroids, anti-hypertensives, contraceptives, anti-depressants, pain killers a.o.; no topical medication affecting the neuro-endocrine systems such as cortisol or nicotine; no reported chronic disease requiring medical attention including obesity, hypertension, metabolic disease, allergic disease, mental disorder; no excessive coffee (more than two cups per day), tea, tobacco or alcohol consumption; no excessive physical activity; no coffee, tea, nicotine or sports on the morning of data collection; no acute infection; not pregnant; no additional severe life-stress during the observation period (e.g., death of a family member); technical issues (e.g., blood lysis contamination). Study participants were aged between 21–32 years (MW 25.91 +/- SD 2.27); had a body mass index between 18–28 (MW 21.39 +/- SD 2.13) (**Table 1**); had blond to light brown natural hair color and mostly shoulder length or longer hair. Most of the study participants lived with other students or family, were unmarried but in a partnership, were socially active and had sufficient financial support from the government or their parents. Study participants generally lived under typical conditions for a student in the city center of Berlin and were mentally healthy [24], [25], [26], [27]. No differences between groups, or between points in time, were found in the analysis of these dimensions. All of the study participants passed the exam, and were satisfied with their level of academic achievement.

**Table 1:** Demographic and self-report mental health baseline characteristics of study participants.

	full sample		exam group		comparison group		
	mean	sd	mean	sd	mean	sd	p
age in years	25.91	*± 2*.*27*	27.00	± 1.00	24.60	± 2.65	**0.00**
BMI	21.32	± 2.19	21.60	± 2.34	20.98	± 1.93	0.43
hours of physical activity per week	1.89	± 2.19	2.03	± 2.02	1.73	± 2.36	0.71
hours of leisure activity per week	5.55	± 4.55	5.67	± 4.52	5.40	± 4.59	0.87
cigarettes per day	0.38	± 1.72	0.67	± 2.29	0.03	± 0.12	0.30
cups of coffee / tea per day	2.55	± 1.74	2.75	± 1.67	2.36	± 1.79	0.57
HADS depression (cut-off 8 out of 21)*	2.64	± 2.41	3.00	± 2.69	2.20	± 1.94	0.36
HADS anxiety (cut-off 8 out of 21)*	5.58	± 3.09	6.39	± 3.30	4.60	± 2.50	0.10
SOMS symptoms (cut-of females 9 out of 53)^#^	8.94	± 6.66	8.22	± 6.14	9.80	± 7.13	0.51
DHUS hassels^$^	3.42	± 2.17	3.11	± 2.54	3.80	± 1.56	0.38
IES sum score^§^	8.79	± 13.17	9.83	± 14.63	7.53	± 11.04	0.63

Note that all participants were female and did not take oral or topical medication affecting the neuro-endocrine systems. P-values refer to comparison between exam (N = 18) and comparison group (N = 15). Significant differences are bold.

*[24],

^#^[25],

^$^[26],

^§^[27].

### Life-stress paradigm and sample size determinants

With respect to the choice of a life-stress paradigm, we considered the expected effects of highly acute experimental paradigms such as the Trier Social Stress Test (TSS) to be too short lived, and too mild to alter hair growth. Highly acute stress paradigms were shown to increase salivary- but not hair-cortisol [28] while prolonged distress paradigms have the opposite effect [29]. We therefor expected major stress to more strongly affect hair biology than highly acute stress paradigms. Populations affected by chronic and severe stress paradigms such as traumatic experience, work related stress or the stress of care giving [30–33] appeared too diverse to meet the requirement of a small, sociobiologically homogeneous pilot sample population. We therefor chose the final exam for German medical students, as a naturalistic paradigm for perceived stress in life [34–37]. This exam requires 12 weeks of focused preparation for the written part, which consists of three consecutive days with 5 hours of multiple-choice testing each day, covering all clinical medical fields. Students have only two attempts to pass this exam. Hence, the students are exposed to exam preparation and performance stress over a prolonged period of time.

**Fig 1:**
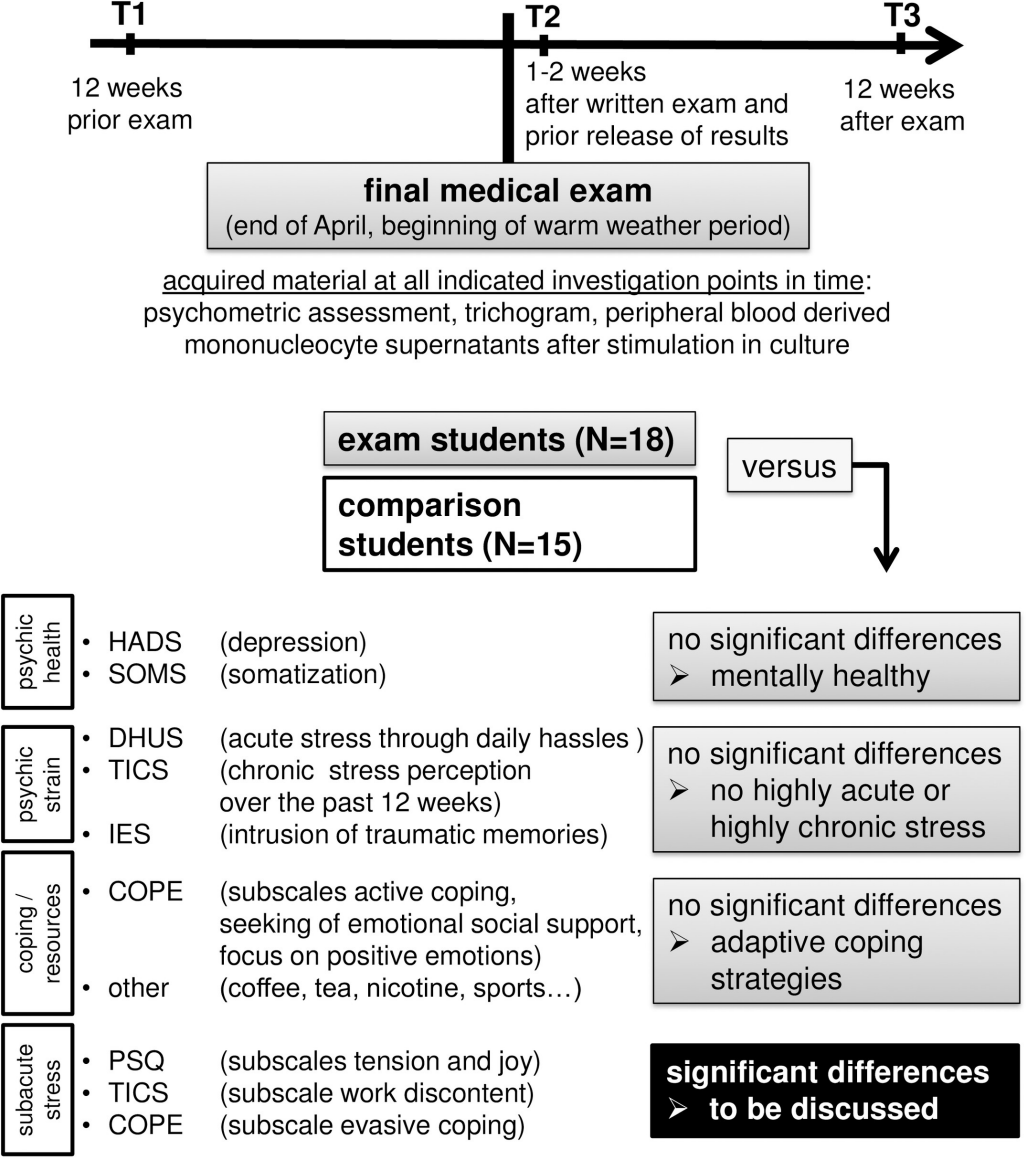
Assessment of stress perception and acquisition of samples to assess biological outcomes in exam versus comparison students. Note that T2 took place within the first two weeks after the written and before the oral part of the exam. A salivary cortisol profile was obtained the day prior to T2 to confirm altered cortisol diurnal profile in the exam group. In addition to assessing subacute stress perception, participants were assessed for mental health, other parameters of psychological strain and coping/resources, to ensure good health and resourcefulness. Abbreviations: **COPE**—coping strategies, **DHUS**—Daily Hassles and Uplifts Scales (Kanner et al., 1981), **HADS**—Hospital Anxiety and Depression Scale, **IES**—Impact of Event Scale, **PSQ**—Perceived Stress Questionnaire, **SOMS**—Screening for Somatoform Symptoms, **TICS**—Trier Inventory for the Assessment of Chronic Stress.

Students participating in the final medical examination are subsequently termed the `exam group´ and compared with students who participated in a regular semester termed the `comparison group´ (**Fig 1**). Minimum required subject numbers of 15 participants per group were calculated by power analysis based on results from other stress studies using comparable outcome measures and reporting at least medium size effects (e.g. [38–42]). A total of 41 students matched the inclusion and exclusion criteria, none of whom dropped out, but 8 had to be excluded from final data analysis due to random presence of exclusion criteria at one of the assessments. This resulted in a final total sample population of 33 study participants, 18 of which were exam and 15 comparison group students.

### Points in time of data acquisition and conditions of sampling

Final medical exams in Germany are scheduled twice a year, one in April and the other in October. In our study, questionnaires, blood samples and hair plucks were collected at three assessment points in time, each 12 weeks apart: T1—January, baseline, twelve weeks prior to the exam and before the learning period started (referred to as baseline or `exam preparation period´); T2—April, within the first and second week after the written part of the exam and before test results were released (referred to as `exam performance period´); T3—July, twelve weeks after the completion of the exam (referred to as `relaxation/regenerative period´). In the comparison group, assessments were conducted in parallel. Participants were monitored for estrous cycle and invited for investigation during identical phases. For each assessment, students were invited to come to our psychoneuroimmunology laboratory in groups of six to eight participants at 8 o´clock in the morning. Blood and hair specimens were taken in a quiet atmosphere 30 min after arrival in the laboratory and 10 min before the study questionnaires were distributed. Salivary samples were obtained the day before T2 under real life conditions as described in detail below. Each participant received 200 Euros upon finalization of participation.

### Assessment of self-reported stress perception

**Fig 2:**
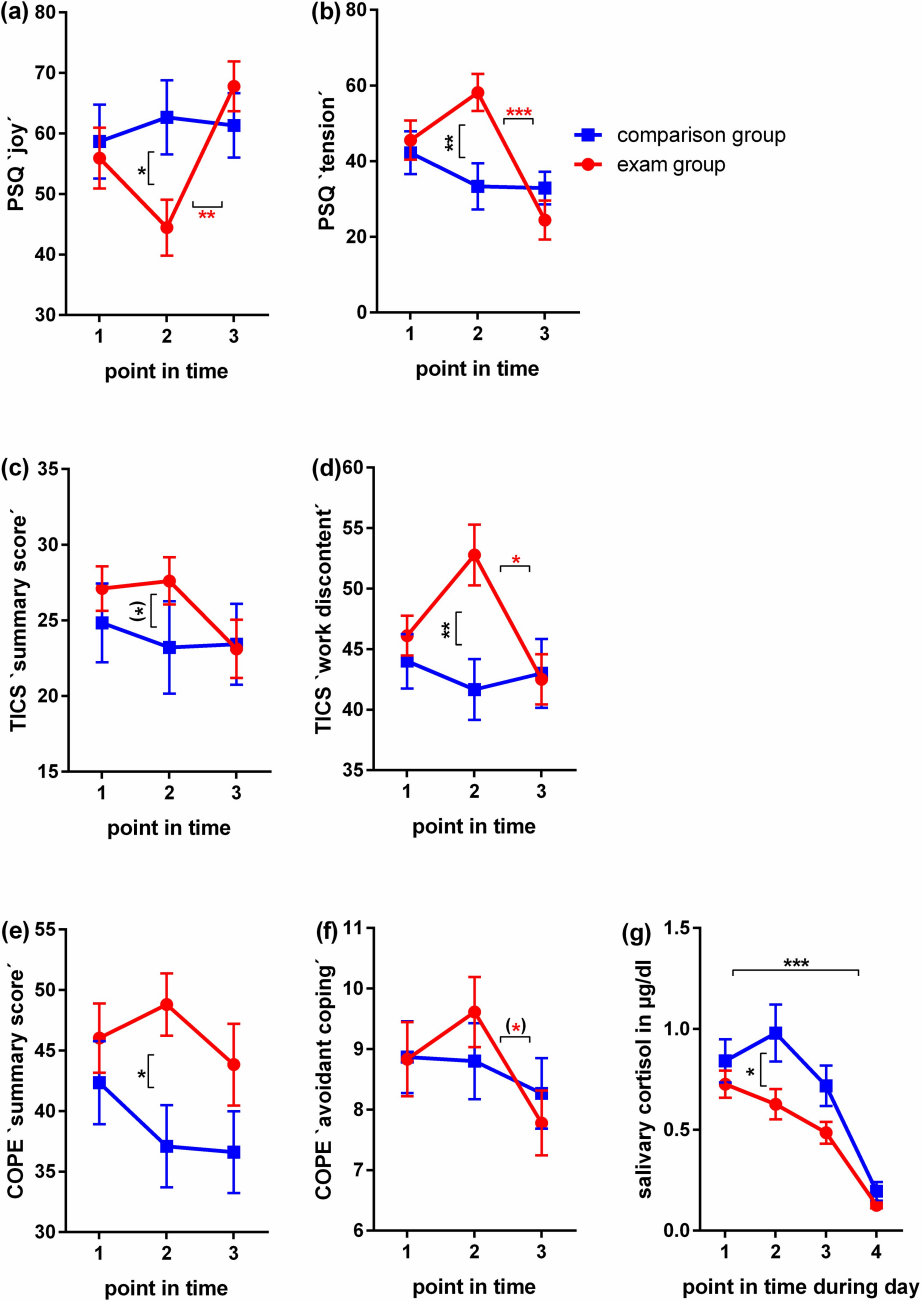
Self report assessment of stress perception and emotional strain in female medical students during their final exam. Graphs (a)-(f) show self-report data assessed at the following points in time (T): baseline, twelve weeks prior to the exam and before the learning period started (1); within the first and second week after the written part of the exam and before test results were released (2); and twelve weeks after the completion of the exam (3). Graph (g) shows cortisol measurements in saliva samples taken on the day prior to T2. Samples were processed to assess diurnal cortisol secretion as described below in the Materials and Methods section. (a)-(g): N = 18 in exam group, N = 15 in comparison group. Mean values and SEM are shown. Mann Whitney U tests were used to examine differences between group means at distinct points in time, Kruskal-Wallis tests with post-hoc Dunn's tests corrected for multiple comparisons to examine differences between different points in time within one group. P-values < 0.1 –one asterix in brackets, <0.05 –one asterix, < 0.01—two asterix, < 0.001—three asterix.

All questionnaires employed were administered in German, using the versions as abbreviated below and presented to all participants at all investigation points in time as indicated in **Fig 1 and Fig 2**. We used: the ‘Daily Hassles and Uplifts Scales’ (DHUS) [43, 44] to assess highly acute stress perception; two subscales of the ‘Perceived Stress Questionnaire’ (PSQ), a questionnaire frequently used for the assessment of stress-hypersensitivity in individuals with medical conditions [45–48], to address more (sub)acute stress-related modulations of mood and wellbeing (`tension´ and `joy´) over the four weeks preceding the assessment [45, 49]; the sum score and a subscale of the ‘Trier Inventory for the Assessment of Chronic Stress’ (TICS) to assess chronic stress and work-strain perception over the 12 weeks preceding the assessment [50, 51]; the ‘COPE’ for the assessment of coping strategies and resources [52]; the ‘Impact of Event Scale’ (IES) to rule out chronic major stress effects as a result of any traumatizing life experiences [53]; the ‘Hospital Anxiety and Depression Scale’ (HADS) [54] to rule out depression and anxiety values above cut-offs; the ‘Screening for Somatoform Symptoms’ (SOMS) [55] to rule out somatization symptoms. In addition, we included items on basic socio-demographic data such as year of birth, socio-economic status, and health related-issues such as medication.

### Saliva sample processing

As a substantial number of studies have failed to show correlations between simultaneously measured cortisol in blood, saliva and target tissue, we considered single point measurements in blood and saliva unsuitable to describe the prolonged stress elicited by final medical exam preparation and participation. We therefor decided to monitor the diurnal cortisol profile at T2 to assess baseline HPA reactivity during the exam performance period. Saliva was sampled with the help of salivettes (Sarstedt, Rommelsdorf, Germany) on the day before T2. Study participants were instructed to collect four saliva samples: 30 min after getting up, 60 min after getting up, 90 minutes after getting up and in the evening, 4 hrs before going to bed (**Fig 2**). Cortisol was measured using human cortisol ELISA kit for saliva (IBL International®, Hamburg, Germany; minimum detection level 0.003 μg/dl; results are given in μg/dl) following the manufacturer’s instructions and previous publications [56–59].

### Blood sample processing

**Fig 3:**
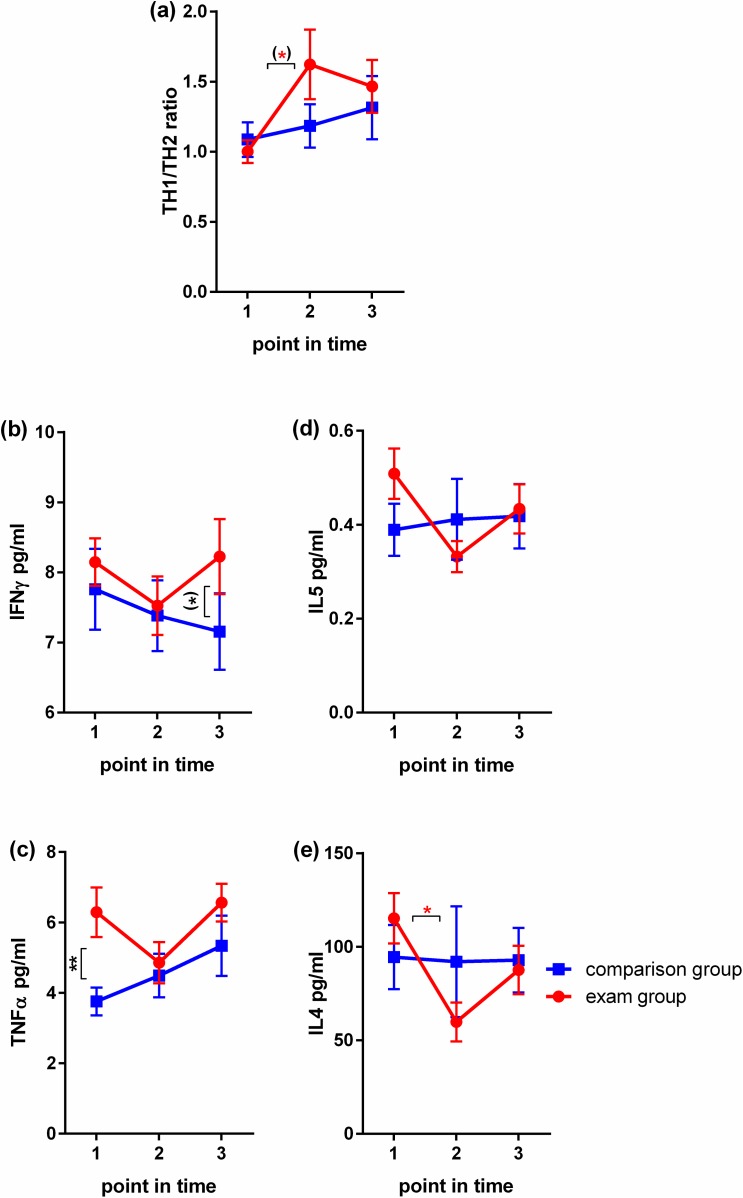
Assessment of immune mediators in female medical students during their final exam. (a)-(e): N = 18 in exam group, N = 15 in comparison group. Mann Whitney U tests were used to examine differences between group means at distinct points in time, Kruskal-Wallis tests with post-hoc Dunn's tests corrected for multiple comparisons to examine differences between different points in time within one group. Mean values and SEM are shown. P-values < 0.1 –one asterix in brackets, <0.05 –one asterix, < 0.01—two asterix.

Cytokines released in response to stress can be detected both in target organs such as the skin, as well as in cells of the peripheral blood [12, 60–62]. As skin sampling is invasive, we chose to sample blood. Blood samples were immediately processed for further Cytometrix Bead Array analysis (CBA; Bender MedSystems, eBioscience, Frankfurt, Germany). Heparinized blood was diluted in sterile PBS (PAA Laboratories GmbH, Pasching, Austria) at room temperature underplayed with Biocoll Separating Solution (Biocoll, Biochrome AG, Berlin, Germany) and centrifuged to obtain peripheral blood mononuclear cells (PBMCs) in the interphase. Cells were diluted to a final concentration of 1.25*106 and stimulated in duplicates with phytohemagglutinin (PHA, lectin from Phaseolus vulgaris; Sigma, Sigma-Aldrich Corporation, St. Louis, MO, USA), PMI (mix of ionomycin and PMA; Sigma) and concanavalin A (ConA, lectin from Canavalia ensiformis = Jack bean; Sigma) or AIM V® (Gibco®, Life Technologies™ GmbH, Darmstadt, Germany). Supernatants were collected and stored at -80°Celsius until further processing. Cytometric Bead Array analysis was performed after completion of sample acquisition using a fluorescent activated cell sorter (FACS, FACSCaliburTM, Becton). The supernatants were thawed and handled as described by the manufacturer (Bender MedSystems). The cytokine concentrations were calculated using FlowCytomixPro™ 2.4 software (Bender MedSystems). The minima/maxima of the cytokine concentrations [pg/ml] were as follows: IL4: 0.000/0.281; IL5: 0.000/1.568; IFNγ: 1.265/16.791; and TNFα: 0.248/35.549. Individual cytokine measurements were recalculated in relation to the mean (individual cytokine measurement = [100/mean of all cytokine measurements in pg/ml]*individual cytokine measurement in pg/ml). The sum of IFNγ and TNFα was than divided by the sum of IL4 and IL5 per participant and point in time; thus, levels above 100 correspond to TH1, and levels below 100 to TH2 response as shown in **Fig 3**.

### Trichogram acquisition and evaluation

The 12 weeks between the investigation times, matched not only with the academic schedule but also with the naturally occurring chronobiological dynamics of hair growth. The following considerations apply: the hair cycle is a model system for the study of tissue regeneration processes in general. HF continuously cycle through stages of regression of the entire hair producing apparatus (catagen), relative quiescence (telogen) and it´s regeneration (anagen). In healthy humans under regular life conditions approximately 10% of all scalp HF are in telogen, while 80–90% are in anagen and equipped with actively pigment producing melanocytes [63–65]. A rise in telogen percentage of up to 30% was reported e.g. in response to biological stressors such as surgery or acute inflammation [5] but also in monkeys exposed to sever housing stress [66]. In murine synchronized hair growth, psycho-social stress was shown to increase the telogen rate [67] by provoking premature catagen [68], whereby the length of the prematurely induced regression phase and the following telogen was not affected. Catagen in humans lasts for two to three weeks and telogen for approximately 10–12 weeks. If the length of these phases is as fixed in humans as it is in mice, morphologically discernible catagen and telogen induction in trichogram analysis in humans can be expected to occur within weeks after stress-exposure while full recovery of hair growth can be expected approximately 12 weeks after induction of regression.

To collect hair tips for respective analysis, all participants were asked not to wash and comb their hair for 3 days before the assessment, in order not to accidentally remove loose hair. Approximately 100 hairs per participant and assessment point in time were plucked according to previously published routine trichogram protocols [63, 69]. Briefly, a row of hairs from the temporal scalp, where hair growth is hormone-independent, was fastened by the distal end with a rubber coated surgical clamp always in the same location. It was than plucked in one swift movement, a procedure which leaves no visible traces and only induces a brief pain, which subsides within seconds after plucking and is usually well tolerated. Plucking was done by or under supervision of an experienced dermatologist (EP), and care was taken to minimize pulling artifacts. It is generally recommended the sampling procedure be performed by skilled and well trained personnel.

**Fig 4:**
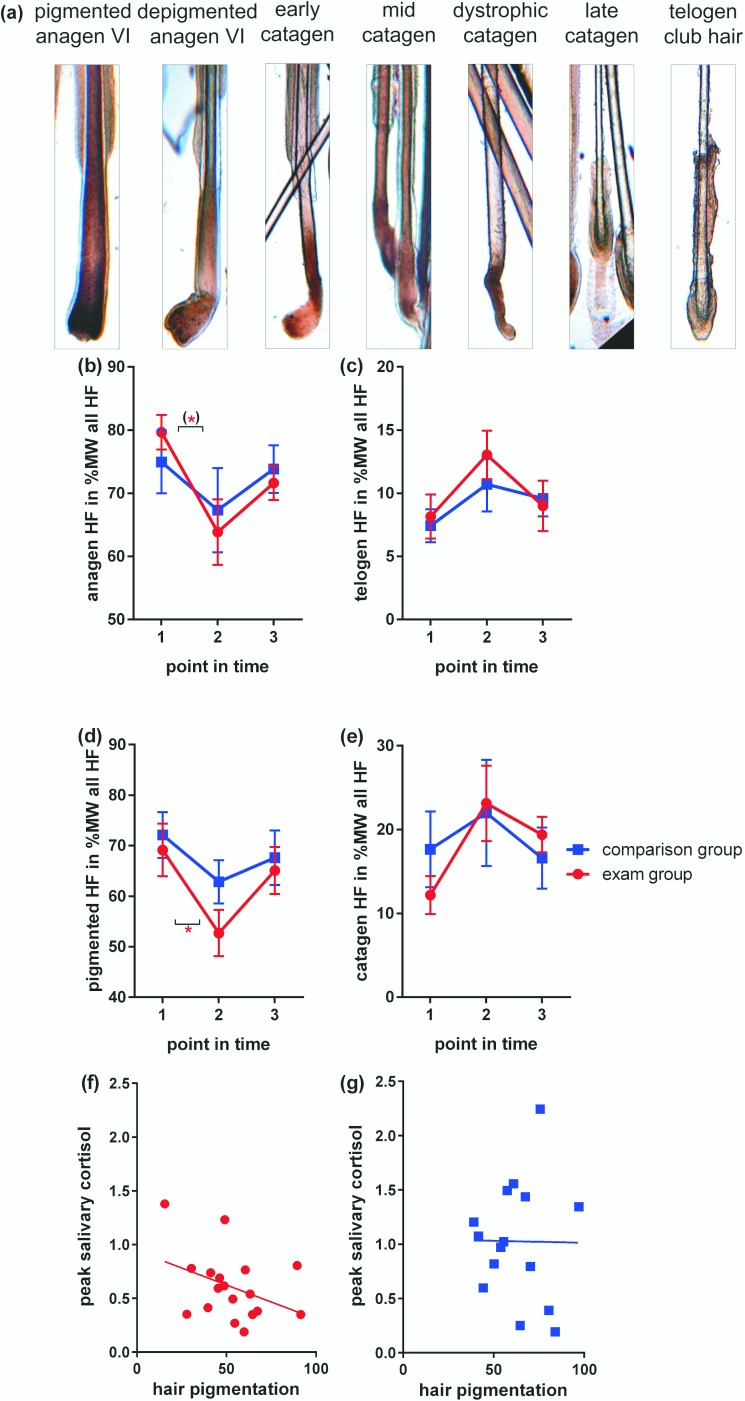
Assessment of hair cycle progression and pigmentation in female medical students during their final exam. (a): representative photomicrographs illustrate morphology of HF in the respective hair cycle and pigmentation state. (b)-(e): N = 18 in exam group, N = 15 in comparison group. Mann Whitney U tests were used to examine differences between group means at distinct points in time, Kruskal-Wallis tests with post-hoc Dunn's tests corrected for multiple comparisons to examine differences between different points in time within one group. Mean values and SEM are shown. P-values < 0.1 –one asterix in brackets, <0.05 –one asterix. Note that some seasonal fluctuations can be observed in the comparison group students. (f) and (g) Pearson correlation of hair pigmentation at T2 with peak salivary cortisol level (point in time 2) shown in **Fig 2** in exam group participants (f) and comparison group participants (g).

Analyses of hair cycle stage and hair pigmentation were performed by two independent investigators on digital photographs taken with a MOTIC BA 400 (Motic, Wetzlar, Germany) at 200-fold magnification. By plucking hair, the tip comes from the follicle with its associated sheaths (inner and outer root sheath) and part of the hair bulb, but leaves the bulk of the proximal hair bulb in the skin. For hair cycle staging, hair tips were classified as in anagen, if a vigorous thick tip was attached to the plucked hair according to standardized classification protocols (e.g. [65, 70]) and as shown in **Fig 4**. Hair tips were classified as in telogen, if the classical club hair appearance was present. Hair tips that showed neither anagen nor telogen morphology were considered to represent mainly catagen/dystrophic HF. Hair tips with a classical club hair morphology were classified as telogen. Based on the number of HF present in the individual total hair sample, the percentages of anagen, telogen and catagen HF were calculated to assess the degree of tissue regeneration capacity in the participant.

As the plucked hair tip is normally hidden inside the scalp skin, it is not affected by hair dyes. Reduced pigmentation of plucked hair tips is present when HF enter the transgression from anagen to catagen. This can serve as a very early indicator of HF regression prior to other morphologically discernible changes of the hair tip [71] and long before the HF has ceased to produce a hair shaft and the characteristic telogen club hair morphology can be observed. To monitor cessation of pigment production at the onset of HF regression, pigmentation was therefore studied in addition to hair cycle staging, as described previously. Analysis was done by histomorphometric assessment, recording fully pigmented HF as such and HF with diluted pigmentation as depigmented (**Fig 4**) [72].

### Statistical analysis

For self-reported data, ordinal items were transformed to a scale ranging from 0 (minimum value, e.g., "do not agree at all") to 100 points (maximum value, e.g., "fully agree"), where applicable. The category "does not apply" and item non-response were coded as missing values which were treated as such and no imputation techniques were used. Calculation of the TH1/TH2 ratio was done as described previously [46, 73]. Statistical analyses were conducted using the IBM SPSS Statistics, Version 22.0.0.2 for Windows software (Armonk, NY, USA) or GraphPad Prism for Windows, version 6.05 (GraphPad Software, La Jolla, CA, USA, www.graphpad.com). All values are shown as the mean and standard deviation (SD) per group and point in time. We expected stress perception and biological measures to differ between groups during exam performance (T2). We also expected stress perception and biological measures to change significantly from baseline (T1) to T2 and from T2 to relaxation (T3) in the experimental group but not in the comparison group. After testing for outliers and normal distribution, the following analyses were performed to confirm these hypothesis: Mann Whitney U tests to examine whether there were differences between group means at distinct points in time, and Kruskal-Wallis tests with post-hoc Dunn's tests corrected for multiple comparisons to examine whether there were differences between different points in time within one group. In addition, the hair measure with clearest difference between exam and comparison group at T2 (pigmentation) was correlated to peak salivary cortisol levels (point in time 2). All p-values given were two-tailed, p-values of less than 0.05 were considered significant, p-values between 0.05 and 0.1 are reported as trends. As potential covariates such as sex, age and BMI were controlled for by the study design, these were not included in the analysis. The only exception was age, which differed between the groups by two years because the comparison group students were less advanced in their studies and hence, younger (**Table 1**). Controlling for outliers or age did not change any of the reported results nor did calculation with the datasets of all 41 included participants, which are therefore reported unadjusted and focused on volunteers to which no exclusion criteria applied. All results are shown in the figures and tables.

## Results

In this section we report the observed inter-group and inter-time-point differences for each reported psychological and biological measure comparing healthy female medical students exposed to major exam stress with an unstressed comparison group and comparing non-stress to stress points in time across the following dimensions (**Table 1**, **Fig 1**): a) self-report data (**Fig 2**), b) immune data (**Fig 3**) and c) data on hair cycling and pigmentation (**Fig 4**).

### Exam stress associates with tension, lack of joy and work discontent

Group-wise comparisons at T1 (exam preparation period), T2 (exam performance period) and T3 (relaxation/regenerative period) respectively revealed a significant difference between exam and comparison students at T2 for PSQ `tension´ (higher) and `joy´ (lower) and TICS `work discontent´ (higher) (**Fig 2**). Hence, the exam students report higher stress perception over the 4–12 weeks prior to assessment. In addition, levels of the COPE summary score (lower) showed a trend at T2 (**Fig 2**). Twelve weeks prior to (T1) or after the exam (T3) the two groups did not differ significantly in any of these measures. Pair-wise analysis comparing points in time within the groups revealed a significant change over time from the exam to relaxation (T2 to T3) for `joy´ (rise), `tension´ and `work discontent´ (drop) (**Fig 2**) within the exam group only. We also found a trend in the change from T2 to T3 for COPE `avoidant coping´ (**Fig 2**). COPE `avoidant coping´ and PSQ `tension´ scored lower than the comparison group at T3 (**Fig 2**), indicating a noticeable relief from exam stress 12 weeks after successful participation. No significant time-wise changes were found within the comparison group (**Fig 2**). The German final medical exam therefore proved to be a valid stress paradigm for our study, whereby stress was measured over a prolonged period of time. The measurement at 12 weeks after the exam differed more noticeably from the exam situation than the measurement at 12 weeks before the exam. To verify the presence of prolonged stress exposure at T2 in the neuroendocrine dimension, hypothalamus pituitary adrenal axis (HPA) baseline activity was assessed by monitoring the diurnal salivary cortisol profile. Comparison between exam and comparison group students at T2 revealed a significantly attenuated diurnal salivary cortisol rise in exam students indicating an attenuated HPA activity as reported previously under other prolonged stress conditions [74, 75].

### TH1/TH2 ratio rises in exam students

Group-wise comparison of individual cytokine levels and TH1/TH2 ratio at T1, T2 and T3 respectively detected only one significant difference between the groups, which was a higher level of TNFα at T1 in the exam group, and one trend, which was a higher level IFNγ at T3 in the exam group (**Fig 3**). Pair-wise analysis comparing points in time within the groups detected a trend in the TH1/TH2 ratio rise from T1 to T2 in the exam group only (**Fig 3**). This appeared to depend mainly on the change in TH2 cytokines, as IL4 dropped significantly in this group from T1 to T2 (below the comparison group level) (**Fig 3**). The TH1 cytokines TNFα and IFNγ also showed the lowest levels in exam students at T2 but were above the comparison group levels throughout the study (**Fig 3**). Hence, in the exam group only, we found higher baseline cytokine levels, an overall decrease in cytokine production from T1 to T2 and a concomitant shift in cytokine balance toward predominance of TH1 cytokine expression during exam performance (**Fig 3**).

### Hair growth is diminished in exam students at T2

Group-wise analysis of hair health measures at T1, T2 and T3 respectively failed to detect significant differences between exam and comparison group students at any of the three investigation points in time (**Fig 4**). However, pair-wise within group analysis between points in time revealed a significant change over time in the percentage of pigmented HF from T1 to T2 in the exam group only (**Fig 4**). In addition, in the exam group only, we found a trend in the change over time from T1 to T2 for anagen HF, with a percentage lower than in the comparison group at T2 (**Fig 4**). Also, correlation of hair pigmentation at T2 with peak salivary cortisol measurement revealed a negative, albeit not significant (Pearson r = -0.387, p = 0.113) correlation between peak salivary cortisol and hair pigmentation in the exam group only, while no correlation was seen in the comparison group (Pearson r = 0.010, p = 0.971). Hence, only in the exam group, did we detect a significant decrease in hair pigmentation and anagen HF percentage between the stress and non-stress measurement points and some association between stress indicators and hair health at T2.

## Discussion

To our knowledge, this is the first study of life-stress and hair biology in a healthy human sample. We observed changes in the stress perception in a young Caucasian female pilot study population, which indicate prolonged stress exposure, as students prepared for and participated in the final medical exam. Within the exam group, this was accompanied by significant fluctuations in TH1/TH2 cytokine balance and hair measures when comparing T1 or T3 (12 weeks prior or after the exam) with T2 (exam): cytokine balance was significantly tilted and hair growth hampered in the exam group. However, no significant group difference in biological measures could be found at T2 between exam and comparison group students. This suggests that stress-induced hair growth changes are present in healthy humans exposed to major exam stress and coincide with relevant cytokine-balance changes. However, these changes are subtle and fully reversible, and therefore not pathologic.

Our sample population was homogeneous with respect to sociobiological status, which facilitated data acquisition and analysis. However, there are limitations in the study related to: the small sample size; the focus on one sex; inclusion of only healthy individuals exposed to a relatively mild stress; the relatively small number (three) of assessments over the course of 24 weeks; the lack of biopsies for hair cycle staging; the limited number of neuroendocrine-immune stress measurements; and the choice of a quasi-experimental naturalistic study design rather than a random-control study design. Replication of these pilot observations is therefore required for example: in more diverse study populations; in individuals exposed to more severe stress such as traumatic experiences; or with additional outcomes such as morning serum levels of HPA activity markers like cortisol and corticotropin releasing hormone (CRH), neuropeptides like substance P (SP) or neurotrophins like nerve growth factor (NGF), all of which link with hair biology [76–79]. This could confirm their validity for the clinical diagnosis of stress-induced hair loss, as well as for the assessment of biological stress-effects in respective studies. However, measurement of stress mediators in blood provides only limited information on local release and function in skin and a correlation of these measurements with locally produced stress mediators need not exist as shown for example by comparison of hair, saliva and blood cortisol [28, 29, 80]. A trichogram can be easily and non-invasively obtained under field conditions and gives evidence of the local stress impact as done in other studies by the assessment of itch perception [81]. It can be implemented in clinical diagnostic procedures as well as experimental settings without excess equipment or time and money consuming procedures. Done repeatedly at times with high life-stress versus low life-stress, it can be instructive in the clinical diagnosis of stress-induced hair loss as well as in studies on major life-stress, especially when combined with self-report assessments of stress perception and immunological outcomes.

To discuss validity and suitability of the three addressed dimensions (stress perception, immune response change, hair growth and pigmentation) for clinical application and stress studies in more detail, the results demonstrate that the self-report questionnaires employed in the study detect prolonged perceived stress present over 4–12 weeks in healthy humans participating in a major exam. To the authors’ knowledge these instruments have not yet been reported in studies on exam stress and its neuroendocrine-immune effects or in studies on skin and hair biology. The observed significant changes in PSQ `tension´ and `joy´, TICS `work discontent´ and COPE `avoidant coping´ clearly establish that the final medical exam in Germany is a major naturalistic life-stress exposure, a notion strengthened by using measurement points well outside the stress exposure period for comparison.

The study also confirms that *ex vivo* stimulated blood derived cells of the immune system can afford a useful tool to obtain surrogate parameters for biologically relevant immune response changes under real life-stress [82, 83]. However, caution is required with respect to the points in time chosen for investigation. The here reported change in TH1/TH2 balance in the presence of an overall depression of cytokine production was observed from T1 to T2, 12 weeks apart. This shift is consistent with a change in cytokine profile in support of cellular adaptive immunity. At the same time an overall suppression of cytokine production was observed. Studies assessing cytokines minutes to hours around acute exam stress or stress mediator exposure also reported a shift to TH1, often associated with an increase in cytokine production [84–87]. By contrast, some publications, which compared stress-to-control assessments a few days and to up to six weeks before the exam, reported a decrease in cellular adaptive immunity (e.g. [85, 88–93]). Hence, immune changes related to stress follow complex time patterns and require multiple assessments.

Of the hair parameters assessed, hair pigmentation is the earliest indicator of hair growth termination in response to damage in a number of experimental set-ups, such as chemotherapy induced hair loss [94, 95]. Decreased hair tip pigmentation detected in exam students at T2 therefore appears to be a promising surrogate indicator of hampered hair growth in a stressed healthy human. In addition, it may indicate oxidative stress [72] and the onset of premature graying [96]. Some changes in hair measures were however also observed in the comparison group between T1, T2 and T3 and no T2 group differences were found. These observations require careful consideration: the comparison group students were in a regular semester, which ended at T2, hence some end of term stress may have occurred. However, the self-report instruments employed to monitor stress perception did not record changes in this dimension. Another possible explanation for the observed variation is that the 24 week long observation period covered seasonal change. Changes in hair growth of around 10% were reported in the literature during seasonal change from winter (as at T1) to spring (as at T2) [64, 97]. This idea is further supported by the observation that cytokines also showed some fluctuation in the comparison group and cellular immune responses to stress can be altered, when the length of days increases [98]. The study may therefore be underpowered for group by time interaction analysis of stress effects superimposed with seasonal change effects. However, to avoid the influence of season in this naturalistic setting is difficult as final exams are scheduled by the universities at the end of academic terms, hence always at the same time during seasonal change.

Taken together, in our naturalistic quasi-experimental pilot study, biological measures revealed time-wise changes in female medical students experiencing stress during the final medical exam period. These suggest increased hair growth hampering immune-responses and decreased hair growth during times of high stress. However, group-wise comparisons between exam and comparison group students did not reveal significant differences in these measures in a study extensively controlled for confounders of neuroendocrine-immune interaction (e.g. age, sex, BMI, education, smoking, physical activity, time of day, mental health). Certain circumstances of design in a study of naturalistic stress effects and hair biology in healthy humans did not allow for an increase of the participant number, shorter distances between assessment points in time or adoption of a randomized controlled design. These included: the ethical requirements, the natural dynamics of hair growth, and employment of a valid life-stress paradigm for young women. Taking these limitations into account, microscopic hair biology analysis is an instructive potential tool for non-invasive follow-up on intra-individual subclinical biological changes in response to stress [14, 99]. It also provides clinicians and their patients with the reassuring information that stress-induced hair health changes during stress stay within a physiological range as opposed to hair growth diseases such as alopecia arreata [100] and that they are fully reversible.

## References

[1] Segerstrom SC. Resources, stress, and immunity: an ecological perspective on human psychoneuroimmunology. Annals of behavioral medicine: a publication of the Society of Behavioral Medicine. 2010;40(1):114–25. Epub 2010/06/08. 20526755 10.1007/s12160-010-9195-3

[2] Dhabhar FS. Psychological stress and immunoprotection versus immunopathology in the skin. Clin Dermatol. 2013;31(1):18–30. doi: 10.1016/j.clindermatol.2011.11.003 23245970 10.1016/j.clindermatol.2011.11.003

[3] Slavish DC, Graham-Engeland JE, Smyth JM, Engeland CG. Salivary markers of inflammation in response to acute stress. Brain, behavior, and immunity. 2015;44C:253–69. Epub 2014/09/11. PubMed Central PMCID: PMC4275319. 10.1016/j.bbi.2014.08.008 PMC4275319 25205395

[4] Hadshiew IM, Foitzik K, Arck PC, Paus R. Burden of hair loss: stress and the underestimated psychosocial impact of telogen effluvium and androgenetic alopecia. J Invest Dermatol. 2004;123(3):455–7. doi: 10.1111/j.0022-202X.2004.23237.x 15304082 10.1111/j.0022-202X.2004.23237.x

[5] Grover C, Khurana A. Telogen effluvium. Indian J Dermatol Venereol Leprol. 2013;79(5):591–603. doi: 10.4103/0378-6323.116731 23974577 10.4103/0378-6323.116731

[6] Plikus MV, Van Spyk EN, Pham K, Geyfman M, Kumar V, Takahashi JS, et al The Circadian Clock in Skin: Implications for Adult Stem Cells, Tissue Regeneration, Cancer, Aging, and Immunity. J Biol Rhythms. 2015. Epub 2015/01/16. 10.1177/0748730414563537 PMC4441597 25589491

[7] Hunter HJ, Momen SE, Kleyn CE. The impact of psychosocial stress on healthy skin. Clinical and experimental dermatology. 2015;40(5):540–6. doi: 10.1111/ced.12582 25808947 10.1111/ced.12582

[8] Peters EM, Liezmann C, Klapp BF, Kruse J. The neuroimmune connection interferes with tissue regeneration and chronic inflammatory disease in the skin. Annals of the New York Academy of Sciences. 2012;1262(1):118–26. Epub 2012/07/25. 22823443 10.1111/j.1749-6632.2012.06647.x

[9] Nahm M, Navarini AA, Kelly EW. Canities subita: a reappraisal of evidence based on 196 case reports published in the medical literature. Int J Trichology. 2013;5(2):63–8. Epub 2014/01/10. PubMed Central PMCID: PMC3877474. doi: 10.4103/0974-7753.122959 24403766 10.4103/0974-7753.122959 PMC3877474

[10] Theoharides TC. Neuroendocrinology of mast cells: Challenges and Controversies. Experimental dermatology. 2017. 10.1111/exd.13288 28094875

[11] Peters EM, Kuhlmei A, Tobin DJ, Muller-Rover S, Klapp BF, Arck PC. Stress exposure modulates peptidergic innervation and degranulates mast cells in murine skin. Brain, behavior, and immunity. 2005;19(3):252–62. doi: 10.1016/j.bbi.2004.08.005 15797314 10.1016/j.bbi.2004.08.005

[12] Theoharides TC, Alysandratos KD, Angelidou A, Delivanis DA, Sismanopoulos N, Zhang B, et al Mast cells and inflammation. Biochimica et biophysica acta. 2012;1822(1):21–33. Epub 2010/12/28. PubMed Central PMCID: PMC3318920. doi: 10.1016/j.bbadis.2010.12.014 21185371 10.1016/j.bbadis.2010.12.014 PMC3318920

[13] Huang WY, Huang YC, Huang KS, Chan CC, Chiu HY, Tsai RY, et al Stress-induced premature senescence of dermal papilla cells compromises hair follicle epithelial-mesenchymal interaction. Journal of dermatological science. 2017. 10.1016/j.jdermsci.2017.01.003 28117106

[14] Arck PC, Slominski A, Theoharides TC, Peters EM, Paus R. Neuroimmunology of stress: skin takes center stage. J Invest Dermatol. 2006;126(8):1697–704. PubMed Central PMCID: PMC2232898. doi: 10.1038/sj.jid.5700104 16845409 10.1038/sj.jid.5700104 PMC2232898

[15] Wang L, Guo LL, Wang LH, Zhang GX, Shang J, Murao K, et al Oxidative stress and substance P mediate psychological stress-induced autophagy and delay of hair growth in mice. Arch Dermatol Res. 2014. Epub 2014/12/17. 10.1007/s00403-014-1521-3 25501647

[16] Pace TW, Heim CM. A short review on the psychoneuroimmunology of posttraumatic stress disorder: from risk factors to medical comorbidities. Brain, behavior, and immunity. 2011;25(1):6–13. doi: 10.1016/j.bbi.2010.10.003 20934505 10.1016/j.bbi.2010.10.003

[17] Eyre H, Baune BT. Neuroplastic changes in depression: a role for the immune system. Psychoneuroendocrinology. 2012;37(9):1397–416. doi: 10.1016/j.psyneuen.2012.03.019 22525700 10.1016/j.psyneuen.2012.03.019

[18] Madva EN, Granstein RD. Nerve-derived transmitters including peptides influence cutaneous immunology. Brain, behavior, and immunity. 2013;34:1–10. Epub 2013/03/23. PubMed Central PMCID: PMC3750093. doi: 10.1016/j.bbi.2013.03.006 23517710 10.1016/j.bbi.2013.03.006 PMC3750093

[19] Marucha PT, Kiecolt-Glaser JK, Favagehi M. Mucosal wound healing is impaired by examination stress. Psychosomatic medicine. 1998;60(3):362–5. 9625226 10.1097/00006842-199805000-00025

[20] Roy S, Khanna S, Yeh PE, Rink C, Malarkey WB, Kiecolt-Glaser J, et al Wound site neutrophil transcriptome in response to psychological stress in young men. Gene Expr. 2005;12(4–6):273–87. Epub 2005/12/20. 16358416 10.3727/000000005783992025 PMC6009119

[21] Mavros MN, Athanasiou S, Gkegkes ID, Polyzos KA, Peppas G, Falagas ME. Do psychological variables affect early surgical recovery? PloS one. 2011;6(5):e20306 Epub 2011/06/03. PubMed Central PMCID: PMC3102096. doi: 10.1371/journal.pone.0020306 21633506 10.1371/journal.pone.0020306 PMC3102096

[22] Sharpley CF, McFarlane JR, Slominski A. Stress-linked cortisol concentrations in hair: what we know and what we need to know. Reviews in the neurosciences. 2011. Epub 2011/12/14. 10.1515/RNS.2011.058 PMC3381079 22150070

[23] Bruenahl CA, Arck PC, Linden M. Limitation of pro- and anti-inflammatory cytokine analysis to discriminate biological stress effects in patients suffering from chronic psychological distress. Nord J Psychiatry. 2012;67(3):191–6. Epub 2012/07/24. doi: 10.3109/08039488.2012.700947 22817281 10.3109/08039488.2012.700947

[24] Bjelland I, Dahl AA, Haug TT, Neckelmann D. The validity of the Hospital Anxiety and Depression Scale. An updated literature review. Journal of psychosomatic research. 2002;52(2):69–77. 11832252 10.1016/s0022-3999(01)00296-3

[25] Fritzsche K, Burger T, Hartmann A, Nubling M, Spahn C. The psychosocial evaluation of medically-ill inpatients—accordance between mental disorders and self-rated psychosocial distress. Psychosoc Med. 2005;2:Doc11 PubMed Central PMCID: PMC2736493. 19742063 PMC2736493

[26] Filipp SH, Ahammer I, Angleitner A, Olbrich E. Eine Untersuchung zu inter- und intraindividuellen Differenzen in der Wahrnehmung und Verarbeitung von subjektiv erlebten Persönlichkeitsveränderungen. Forschungsbericht Nr 11 aus dem Projekt Entwicklungspsychologie des Erwachsenenalters. Trier: Universität, Fachbereich I—Psychologie; 1980.

[27] Horowitz M, Wilner N, Alvarez W. Impact of Event Scale: A measure of subjective stress. Psychosomatic medicine. 1979;(41):209–18. 472086 10.1097/00006842-197905000-00004

[28] Grass J, Kirschbaum C, Miller R, Gao W, Steudte-Schmiedgen S, Stalder T. Sweat-inducing physiological challenges do not result in acute changes in hair cortisol concentrations. Psychoneuroendocrinology. 2015;53:108–16. doi: 10.1016/j.psyneuen.2014.12.023 25615913 10.1016/j.psyneuen.2014.12.023

[29] Steudte S, Stalder T, Dettenborn L, Klumbies E, Foley P, Beesdo-Baum K, et al Decreased hair cortisol concentrations in generalised anxiety disorder. Psychiatry research. 2011;186(2–3):310–4. doi: 10.1016/j.psychres.2010.09.002 20889215 10.1016/j.psychres.2010.09.002

[30] Gouin JP, Glaser R, Malarkey WB, Beversdorf D, Kiecolt-Glaser J. Chronic stress, daily stressors, and circulating inflammatory markers. Health psychology: official journal of the Division of Health Psychology, American Psychological Association. 2012;31(2):264–8. PubMed Central PMCID: PMC3253267. 10.1037/a0025536 PMC3253267 21928900

[31] Gouin JP, Glaser R, Malarkey WB, Beversdorf D, Kiecolt-Glaser JK. Childhood abuse and inflammatory responses to daily stressors. Annals of behavioral medicine: a publication of the Society of Behavioral Medicine. 2012;44(2):287–92. PubMed Central PMCID: PMC3699690. 22714139 10.1007/s12160-012-9386-1 PMC3699690

[32] Brunet A, Thomas E, Saumier D, Ashbaugh AR, Azzoug A, Pitman RK, et al Trauma reactivation plus propranolol is associated with durably low physiological responding during subsequent script-driven traumatic imagery. Can J Psychiatry. 2014;59(4):228–32. Epub 2014/07/10. PubMed Central PMCID: PMC4079131. doi: 10.1177/070674371405900408 25007116 10.1177/070674371405900408 PMC4079131

[33] Gidlow CJ, Randall J, Gillman J, Silk S, Jones MV. Hair cortisol and self-reported stress in healthy, working adults. Psychoneuroendocrinology. 2016;63:163–9. doi: 10.1016/j.psyneuen.2015.09.022 26447679 10.1016/j.psyneuen.2015.09.022

[34] Kamezaki Y, Katsuura S, Kuwano Y, Tanahashi T, Rokutan K. Circulating cytokine signatures in healthy medical students exposed to academic examination stress. Psychophysiology. 2012;49(7):991–7. doi: 10.1111/j.1469-8986.2012.01371.x 22468981 10.1111/j.1469-8986.2012.01371.x

[35] Zunhammer M, Eberle H, Eichhammer P, Busch V. Somatic symptoms evoked by exam stress in university students: the role of alexithymia, neuroticism, anxiety and depression. PloS one. 2013;8(12):e84911 PubMed Central PMCID: PMC3867544. doi: 10.1371/journal.pone.0084911 24367700 10.1371/journal.pone.0084911 PMC3867544

[36] Wolfram M, Bellingrath S, Feuerhahn N, Kudielka BM. Cortisol responses to naturalistic and laboratory stress in student teachers: comparison with a non-stress control day. Stress Health. 2013;29(2):143–9. doi: 10.1002/smi.2439 22888074 10.1002/smi.2439

[37] Merz CJ, Wolf OT. Examination of cortisol and state anxiety at an academic setting with and without oral presentation. Stress. 2015;18(1):138–42. doi: 10.3109/10253890.2014.989206 25407296 10.3109/10253890.2014.989206

[38] Peters EM, Arck PC, Paus R. Hair growth inhibition by psychoemotional stress: a mouse model for neural mechanisms in hair growth control. Experimental dermatology. 2006;15(1):1–13. doi: 10.1111/j.0906-6705.2005.00372.x 16364026 10.1111/j.0906-6705.2005.00372.x

[39] Peters EM, Liotiri S, Bodo E, Hagen E, Biro T, Arck PC, et al Probing the effects of stress mediators on the human hair follicle: substance P holds central position. The American journal of pathology. 2007;171(6):1872–86. doi: 10.2353/ajpath.2007.061206 18055548 10.2353/ajpath.2007.061206 PMC2111110

[40] Koh KB, Lee YJ, Beyn KM, Chu SH, Kim DM. Counter-stress effects of relaxation on proinflammatory and anti-inflammatory cytokines. Brain, behavior, and immunity. 2008;22(8):1130–7. doi: 10.1016/j.bbi.2008.06.009 18639628 10.1016/j.bbi.2008.06.009

[41] Kiecolt-Glaser JK, Belury MA, Andridge R, Malarkey WB, Glaser R. Omega-3 supplementation lowers inflammation and anxiety in medical students: A randomized controlled trial. Brain, behavior, and immunity. 2011;25(8):1725–34. Epub 2011/07/26. PubMed Central PMCID: PMC3191260. doi: 10.1016/j.bbi.2011.07.229 21784145 10.1016/j.bbi.2011.07.229 PMC3191260

[42] Paine NJ, Ring C, Aldred S, Bosch JA, Wadley AJ, Veldhuijzen van Zanten JJ. Eccentric-exercise induced inflammation attenuates the vascular responses to mental stress. Brain, behavior, and immunity. 2013. Epub 2013/02/05. 10.1016/j.bbi.2013.01.082 23376168

[43] Hartman JM, Berger A, Baker K, Bolle J, Handel D, Mannes A, et al Quality of life and pain in premenopausal women with major depressive disorder: the POWER Study. Health Qual Life Outcomes. 2006;4:2 Epub 2006/01/20. PubMed Central PMCID: PMC1373611. doi: 10.1186/1477-7525-4-2 16420706 10.1186/1477-7525-4-2 PMC1373611

[44] Kanner AD, Coyne JC, Schaefer C, Lazarus RS. Comparison of two modes of stress measurement: daily hassles and uplifts versus major life events. J Behav Med. 1981;4(1):1–39. Epub 1981/03/01. 7288876 10.1007/BF00844845

[45] Fliege H, Rose M, Arck P, Walter OB, Kocalevent RD, Weber C, et al The Perceived Stress Questionnaire (PSQ) reconsidered: validation and reference values from different clinical and healthy adult samples. Psychosomatic medicine. 2005;67(1):78–88. Epub 2005/01/28. doi: 10.1097/01.psy.0000151491.80178.78 15673628 10.1097/01.psy.0000151491.80178.78

[46] Krohn M, Listing M, Tjahjono G, Reisshauer A, Peters E, Klapp BF, et al Depression, mood, stress, and Th1/Th2 immune balance in primary breast cancer patients undergoing classical massage therapy. Support Care Cancer. 2011;19(9):1303–11. Epub 2010/07/21. doi: 10.1007/s00520-010-0946-2 20644965 10.1007/s00520-010-0946-2

[47] Nordin S, Ljungberg JK, Claeson AS, Neely G. Stress and odor sensitivity in persons with noise sensitivity. Noise & health. 2013;15(64):173–7. Epub 2013/05/22. 23689299 10.4103/1463-1741.112366

[48] Dur M, Sadlonova M, Haider S, Binder A, Stoffer M, Coenen M, et al Health determining concepts important to people with Crohn's disease and their coverage by patient-reported outcomes of health and wellbeing. J Crohns Colitis. 2014;8(1):45–55. Epub 2013/02/05. PubMed Central PMCID: PMC3889494. doi: 10.1016/j.crohns.2012.12.014 23375212 10.1016/j.crohns.2012.12.014 PMC3889494

[49] Levenstein S, Prantera C, Varvo V, Scribano ML, Berto E, Luzi C, et al Development of the Perceived Stress Questionnaire: A new tool for psychosomatic research. Journal of psychosomatic research. 1993;37:19–32. 10.1016/0022-3999(93)90120-5 8421257

[50] Schulz P, Schlotz W. [Trierer Inventar zur Erfassung von chronischem Streß (TICS): Skalenkonstruktion, teststatistische Überprüfung und Validierung der Skala Arbeitsüberlastung]. Diagnostica. 1999;45(1):8–19.

[51] van Leeuwen N, Bellingrath S, de Kloet ER, Zitman FG, DeRijk RH, Kudielka BM, et al Human mineralocorticoid receptor (MR) gene haplotypes modulate MR expression and transactivation: implication for the stress response. Psychoneuroendocrinology. 2011;36(5):699–709. Epub 2010/11/26. doi: 10.1016/j.psyneuen.2010.10.003 21095064 10.1016/j.psyneuen.2010.10.003

[52] Carver CS. You want to measure coping but your protocol's too long: Consider the Brief COPE. International journal of behavioral medicine. 1997;4:92–100. doi: 10.1207/s15327558ijbm0401_6 16250744 10.1207/s15327558ijbm0401_6

[53] Sundin EC, Horowitz MJ. Horowitz's Impact of Event Scale evaluation of 20 years of use. Psychosomatic medicine. 2003;65(5):870–6. Epub 2003/09/26. 14508034 10.1097/01.psy.0000084835.46074.f0

[54] Herrmann C. International experiences with the Hospital Anxiety and Depression Scale—a review of validation data and clinical results. Journal of psychosomatic research. 1997;42(1):17–41. Epub 1997/01/01. 9055211 10.1016/s0022-3999(96)00216-4

[55] Rief W, Hiller W. A new approach to the assessment of the treatment effects of somatoform disorders. Psychosomatics. 2003;44(6):492–8. Epub 2003/11/05. doi: 10.1176/appi.psy.44.6.492 14597684 10.1176/appi.psy.44.6.492

[56] Karlen J, Ludvigsson J, Frostell A, Theodorsson E, Faresjo T. Cortisol in hair measured in young adults—a biomarker of major life stressors? BMC Clin Pathol. 2011;11:12 PubMed Central PMCID: PMC3217842. doi: 10.1186/1472-6890-11-12 22026917 10.1186/1472-6890-11-12 PMC3217842

[57] Gonzalez-de-la-Vara R, Valdez RA, Lemus-Ramirez V, Vazquez-Chagoyan JC, Villa-Godoy A, Romano MC. Effects of adrenocorticotropic hormone challenge and age on hair cortisol concentrations in dairy cattle. Can J Vet Res. 2011;75(3):216–21. PubMed Central PMCID: PMC3122973. 22210998 PMC3122973

[58] Albar WF, Russell EW, Koren G, Rieder MJ, van Umm SH. Human hair cortisol analysis: comparison of the internationally-reported ELISA methods. Clin Invest Med. 2013;36(6):E312–6. 24309228 10.25011/cim.v36i6.20629

[59] Meyer J, Novak M, Hamel A, Rosenberg K. Extraction and analysis of cortisol from human and monkey hair. Journal of visualized experiments: JoVE. 2014;(83):e50882 PubMed Central PMCID: PMC4089402. doi: 10.3791/50882 24513702 10.3791/50882 PMC4089402

[60] Suto H, Nakae S, Kakurai M, Sedgwick JD, Tsai M, Galli SJ. Mast cell-associated TNF promotes dendritic cell migration. Journal of immunology. 2006;176(7):4102–12. 10.4049/jimmunol.176.7.4102 16547246

[61] Asadi S, Alysandratos KD, Angelidou A, Miniati A, Sismanopoulos N, Vasiadi M, et al Substance P (SP) induces expression of functional corticotropin-releasing hormone receptor-1 (CRHR-1) in human mast cells. J Invest Dermatol. 2012;132(2):324–9. Epub 2011/11/18. doi: 10.1038/jid.2011.334 22089831 10.1038/jid.2011.334 PMC3471564

[62] Liu ZQ, Song JP, Liu X, Jiang J, Chen X, Yang L, et al Mast cell-derived serine proteinase regulates T helper 2 polarization. Scientific reports. 2014;4:4649 Epub 2014/04/12. PubMed Central PMCID: PMC3983597. doi: 10.1038/srep04649 24721951 10.1038/srep04649 PMC3983597

[63] Dhurat R, Saraogi P. Hair evaluation methods: merits and demerits. Int J Trichology. 2009;1(2):108–19. Epub 2009/07/01. PubMed Central PMCID: PMC2938572. doi: 10.4103/0974-7753.58553 20927232 10.4103/0974-7753.58553 PMC2938572

[64] Kahn Ch M, Guerrero AR, Cesped MC. [Seasonal variation of trichogram in Chilean subjects]. Rev Med Chil. 2009;137(11):1437–40. Epub 2010/01/26. doi: /S0034-98872009001100004 20098801

[65] Müller-Röver S, Handjiski B, van der Veen C, Eichmüller S, Foitzik K, McKay IA, et al A comprehensive guide for the accurate classification of murine hair follicles in distinct hair cycle stages. J Invest Dermatol. 2001;117(1):3–15. doi: 10.1046/j.0022-202x.2001.01377.x 11442744 10.1046/j.0022-202x.2001.01377.x

[66] Novak MA, Menard MT, El-Mallah SN, Rosenberg K, Lutz CK, Worlein J, et al Assessing significant (>30%) alopecia as a possible biomarker for stress in captive rhesus monkeys (Macaca mulatta). American journal of primatology. 2017;79(1):1–8. PubMed Central PMCID: PMC5055463. 10.1002/ajp.22547 PMC5055463 27008590

[67] Katayama M, Aoki E, Suzuki H, Kawana S. Foot shock stress prolongs the telogen stage of the spontaneous hair cycle in a non-depilated mouse model. Experimental dermatology. 2007;16(7):553–60. doi: 10.1111/j.1600-0625.2007.00558.x 17576234 10.1111/j.1600-0625.2007.00558.x

[68] Arck PC, Handjiski B, Peters EM, Peter AS, Hagen E, Fischer A, et al Stress inhibits hair growth in mice by induction of premature catagen development and deleterious perifollicular inflammatory events via neuropeptide substance P-dependent pathways. The American journal of pathology. 2003;162(3):803–14. doi: 10.1016/S0002-9440(10)63877-1 12598315 10.1016/S0002-9440(10)63877-1 PMC1868104

[69] Braun-Falco O, Heilgemeir GP. Significance of hair root status method. Hautarzt. 1977 28(3):136–9. 856769

[70] Oh JW, Kloepper J, Langan EA, Kim Y, Yeo J, Kim MJ, et al A Guide to Studying Human Hair Follicle Cycling In Vivo. J Invest Dermatol. 2016;136(1):34–44. PubMed Central PMCID: PMC4785090. doi: 10.1038/JID.2015.354 26763421 10.1038/JID.2015.354 PMC4785090

[71] Tobin DJ. The cell biology of human hair follicle pigmentation. Pigment cell & melanoma research. 2011;24(1):75–88. Epub 2010/11/13. 21070612 10.1111/j.1755-148X.2010.00803.x

[72] Peters EM, Liezmann C, Spatz K, Ungethum U, Kuban RJ, Daniltchenko M, et al Profiling mRNA of the graying human hair follicle constitutes a promising state-of-the-art tool to assess its aging: an exemplary report. J Invest Dermatol. 2013;133(5):1150–60. Epub 2012/12/14. doi: 10.1038/jid.2012.462 23235529 10.1038/jid.2012.462

[73] Siedentopf F, Tariverdian N, Rucke M, Kentenich H, Arck PC. Immune status, psychosocial distress and reduced quality of life in infertile patients with endometriosis. Am J Reprod Immunol. 2008;60(5):449–61. Epub 2009/02/25. 19238750 10.1111/j.1600-0897.2008.00644.x

[74] Klaassens ER, Giltay EJ, van Veen T, Veen G, Zitman FG. Trauma exposure in relation to basal salivary cortisol and the hormone response to the dexamethasone/CRH test in male railway employees without lifetime psychopathology. Psychoneuroendocrinology. 2010;35(6):878–86. Epub 2009/12/29. doi: 10.1016/j.psyneuen.2009.11.012 20036072 10.1016/j.psyneuen.2009.11.012

[75] Tomiyama AJ, Dallman MF, Epel ES. Comfort food is comforting to those most stressed: evidence of the chronic stress response network in high stress women. Psychoneuroendocrinology. 2011;36(10):1513–9. Epub 2011/09/13. doi: 10.1016/j.psyneuen.2011.04.005 21906885 10.1016/j.psyneuen.2011.04.005 PMC3425607

[76] Ito N, Ito T, Kromminga A, Bettermann A, Takigawa M, Kees F, et al Human hair follicles display a functional equivalent of the hypothalamic-pituitary-adrenal axis and synthesize cortisol. FASEB J. 2005;19(10):1332–4. doi: 10.1096/fj.04-1968fje 15946990 10.1096/fj.04-1968fje

[77] Paus R, Langan EA, Vidali S, Ramot Y, Andersen B. Neuroendocrinology of the hair follicle: principles and clinical perspectives. Trends in molecular medicine. 2014;20(10):559–70. doi: 10.1016/j.molmed.2014.06.002 25066729 10.1016/j.molmed.2014.06.002

[78] Kwack MH, Lee JH, Seo CH, Kim JC, Kim MK, Sung YK. Dickkopf-1 is involved in dexamethasone-mediated hair follicle regression. Experimental dermatology. 2017. 10.1111/exd.13308 28155238

[79] Xiang L, Sunesara I, Rehm KE, Marshall GD Jr. Hair Cortisol Concentrations Are Associated with Hair Growth Rate. Neuroimmunomodulation. 2017. 10.1159/000455867 28249276

[80] Short SJ, Stalder T, Marceau K, Entringer S, Moog NK, Shirtcliff EA, et al Correspondence between hair cortisol concentrations and 30-day integrated daily salivary and weekly urinary cortisol measures. Psychoneuroendocrinology. 2016;71:12–8. PubMed Central PMCID: PMC4955743. doi: 10.1016/j.psyneuen.2016.05.007 27235635 10.1016/j.psyneuen.2016.05.007 PMC4955743

[81] Schut C, Weik U, Tews N, Gieler U, Deinzer R, Kupfer J. Coping as mediator of the relationship between stress and itch in patients with atopic dermatitis: a regression and mediation analysis. Experimental dermatology. 2015;24(2):148–50. doi: 10.1111/exd.12578 25363422 10.1111/exd.12578

[82] Maes M, Nowak G, Caso JR, Leza JC, Song C, Kubera M, et al Toward Omics-Based, Systems Biomedicine, and Path and Drug Discovery Methodologies for Depression-Inflammation Research. Mol Neurobiol. 2015. 10.1007/s12035-015-9183-5 25934103

[83] House SL. Psychological distress and its impact on wound healing: an integrative review. J Wound Ostomy Continence Nurs. 2015;42(1):38–41. Epub 2014/12/31. doi: 10.1097/WON.0000000000000080 25549307 10.1097/WON.0000000000000080

[84] Matalka KZ. Neuroendocrine and cytokines-induced responses to minutes, hours, and days of mental stress. Neuro Endocrinol Lett. 2003;24(5):283–92. Epub 2003/12/04. 14646999

[85] Kang DH, Fox C. Th1 and Th2 cytokine responses to academic stress. Res Nurs Health. 2001;24(4):245–57. Epub 2001/12/18. 11746056 10.1002/nur.1027

[86] Xiang L, Marshall GD, Jr. Immunomodulatory effects of in vitro stress hormones on FoxP3, Th1/Th2 cytokine and costimulatory molecule mRNA expression in human peripheral blood mononuclear cells. Neuroimmunomodulation. 2011;18(1):1–10. Epub 2010/07/08. doi: 10.1159/000311450 20606488 10.1159/000311450

[87] Deinzer R, Kleineidam C, Stiller-Winkler R, Idel H, Bachg D. Prolonged reduction of salivary immunoglobulin A (sIgA) after a major academic exam. International journal of psychophysiology: official journal of the International Organization of Psychophysiology. 2000;37(3):219–32. Epub 2000/06/20. 10858568 10.1016/s0167-8760(99)00112-9

[88] Trueba AF, Rosenfield D, Oberdorster E, Vogel PD, Ritz T. The effect of academic exam stress on mucosal and cellular airway immune markers among healthy and allergic individuals. Psychophysiology. 2013;50(1):5–14. doi: 10.1111/j.1469-8986.2012.01487.x 23157618 10.1111/j.1469-8986.2012.01487.x

[89] Glaser R, Pearson GR, Bonneau RH, Esterling BA, Atkinson C, Kiecolt-Glaser JK. Stress and the memory T-cell response to the Epstein-Barr virus in healthy medical students. Health psychology: official journal of the Division of Health Psychology, American Psychological Association. 1993;12(6):435–42. Epub 1993/11/01. 10.1037//0278-6133.12.6.435 8293726

[90] Kiecolt-Glaser JK, Marucha PT, Atkinson C, Glaser R. Hypnosis as a modulator of cellular immune dysregulation during acute stress. J Consult Clin Psychol. 2001;69(4):674–82. Epub 2001/09/12. 11550733 10.1037//0022-006x.69.4.674

[91] Hewig J, Schlotz W, Gerhards F, Breitenstein C, Lurken A, Naumann E. Associations of the cortisol awakening response (CAR) with cortical activation asymmetry during the course of an exam stress period. Psychoneuroendocrinology. 2008;33(1):83–91. Epub 2007/11/21. doi: 10.1016/j.psyneuen.2007.10.004 18022766 10.1016/j.psyneuen.2007.10.004

[92] Loft P, Thomas MG, Petrie KJ, Booth RJ, Miles J, Vedhara K. Examination stress results in altered cardiovascular responses to acute challenge and lower cortisol. Psychoneuroendocrinology. 2007;32(4):367–75. Epub 2007/03/31. doi: 10.1016/j.psyneuen.2007.02.004 17395393 10.1016/j.psyneuen.2007.02.004

[93] Steinisch M, Yusuf R, Li J, Stalder T, Bosch JA, Rahman O, et al Work stress and hair cortisol levels among workers in a Bangladeshi ready-made garment factory—Results from a cross-sectional study. Psychoneuroendocrinology. 2014;50:20–7. doi: 10.1016/j.psyneuen.2014.08.001 25199982 10.1016/j.psyneuen.2014.08.001

[94] Hendrix S, Handjiski B, Peters EM, Paus R. A guide to assessing damage response pathways of the hair follicle: lessons from cyclophosphamide-induced alopecia in mice. J Invest Dermatol. 2005;125(1):42–51. doi: 10.1111/j.0022-202X.2005.23787.x 15982301 10.1111/j.0022-202X.2005.23787.x

[95] Paus R. A neuroendocrinological perspective on human hair follicle pigmentation. Pigment cell & melanoma research. 2011;24(1):89–106. Epub 2010/11/27. 21108769 10.1111/j.1755-148X.2010.00808.x

[96] Akin Belli A, Etgu F, Ozbas Gok S, Kara B, Dogan G. Risk Factors for Premature Hair Graying in Young Turkish Adults. Pediatric dermatology. 2016;33(4):438–42. doi: 10.1111/pde.12881 27292443 10.1111/pde.12881

[97] Braig S, Grabher F, Ntomchukwu C, Reister F, Stalder T, Kirschbaum C, et al Determinants of maternal hair cortisol concentrations at delivery reflecting the last trimester of pregnancy. Psychoneuroendocrinology. 2015;52:289–96. doi: 10.1016/j.psyneuen.2014.12.006 25553388 10.1016/j.psyneuen.2014.12.006

[98] Bilbo SD, Dhabhar FS, Viswanathan K, Saul A, Yellon SM, Nelson RJ. Short day lengths augment stress-induced leukocyte trafficking and stress-induced enhancement of skin immune function. Proc Natl Acad Sci U S A. 2002;99(6):4067–72. Epub 2002/03/21. PubMed Central PMCID: PMC122649. doi: 10.1073/pnas.062001899 11904451 10.1073/pnas.062001899 PMC122649

[99] Siebenhaar F, Sharov AA, Peters EM, Sharova TY, Syska W, Mardaryev AN, et al Substance P as an Immunomodulatory Neuropeptide in a Mouse Model for Autoimmune Hair Loss (Alopecia Areata). J Invest Dermatol. 2007;127(6):1489–97. doi: 10.1038/sj.jid.5700704 17273166 10.1038/sj.jid.5700704

[100] Paus R, Arck P. Neuroendocrine perspectives in alopecia areata: does stress play a role? J Invest Dermatol. 2009;129(6):1324–6. Epub 2009/05/13. doi: 10.1038/jid.2009.111 19434088 10.1038/jid.2009.111

